# Pseudo-Label Assisted nnU-Net enables automatic segmentation of 7T MRI from a single acquisition

**DOI:** 10.3389/fnimg.2023.1252261

**Published:** 2023-12-01

**Authors:** Corinne Donnay, Henry Dieckhaus, Charidimos Tsagkas, María Inés Gaitán, Erin S. Beck, Andrew Mullins, Daniel S. Reich, Govind Nair

**Affiliations:** ^1^Translational Neuroradiology Section, National Institute of Neurological Disorders and Stroke (NINDS), National Institutes of Health, Bethesda, MD, United States; ^2^qMRI Core, NINDS, National Institutes of Health, Bethesda, MD, United States; ^3^Department of Neurology, Icahn School of Medicine at Mount Sinai, New York, NY, United States

**Keywords:** brain and lesion segmentation, 7T MRI, deep learning, transfer learning, multiple sclerosis

## Abstract

**Introduction:**

Automatic whole brain and lesion segmentation at 7T presents challenges, primarily from bias fields, susceptibility artifacts including distortions, and registration errors. Here, we sought to use deep learning algorithms (D/L) to do both skull stripping and whole brain segmentation on multiple imaging contrasts generated in a single Magnetization Prepared 2 Rapid Acquisition Gradient Echoes (MP2RAGE) acquisition on participants clinically diagnosed with multiple sclerosis (MS), bypassing registration errors.

**Methods:**

Brain scans Segmentation from 3T and 7T scanners were analyzed with software packages such as FreeSurfer, Classification using Derivative-based Features (C-DEF), nnU-net, and a novel 3T-to-7T transfer learning method, Pseudo-Label Assisted nnU-Net (PLAn). 3T and 7T MRIs acquired within 9 months from 25 study participants with MS (Cohort 1) were used for training and optimizing. Eight MS patients (Cohort 2) scanned only at 7T, but with expert annotated lesion segmentation, was used to further validate the algorithm on a completely unseen dataset. Segmentation results were rated visually by experts in a blinded fashion and quantitatively using Dice Similarity Coefficient (DSC).

**Results:**

Of the methods explored here, nnU-Net and PLAn produced the best tissue segmentation at 7T for all tissue classes. In both quantitative and qualitative analysis, PLAn significantly outperformed nnU-Net (and other methods) in lesion detection in both cohorts. PLAn's lesion DSC improved by 16% compared to nnU-Net.

**Discussion:**

Limited availability of labeled data makes transfer learning an attractive option, and pre-training a nnUNet model using readily obtained 3T pseudo-labels was shown to boost lesion detection capabilities at 7T.

## Introduction

Volumetric segmentation of brain magnetic resonance images (MRI) is now a commonly used post processing step enabling noninvasive quantitative analysis of disease progression in various neurological and neuropsychiatric diseases such as multiple sclerosis (MS), Alzheimer's disease, human immunodeficiency viral infection, and depression (de Leeuw et al., 2005; Traboulsee et al., 2016; Espinoza Oyarce et al., 2020; Mina et al., 2021). Since manual annotation of 3D MRI data is extremely tedious and time-consuming, automated segmentation methods are of great interest for both clinical and research applications. There is a dearth of brain segmentation algorithms from 7T images compared to 3T and lower fields, especially ones that can efficiently segment tissue classes in the whole brain.

High magnetic field strengths have enabled a more detailed understanding of the underlying pathology of MS. Compared to 3T, 7T MRI offers a higher signal-to-noise ratio enabling sub-millimeter resolution, allowing for more sensitive analyses than were previously possible (Pohmann et al., 2016). This has led to a better detection of MS lesions at 7T compared to 3T as well as a greater understanding of the underlying mechanisms of MS, including the role of cortical lesions in cognitive decline, iron deposition and tissue in disease progression (Harrison et al., 2015). The greater structural detail enabled by 7T imaging also allows more nuanced stratification of pathologies such as MS lesions or glioblastomas (Ladd et al., 2018). However, higher magnetic fields present particular challenges for post-processing due to more pronounced radio-frequency field nonuniformities, more susceptibility artifacts (Haast et al., 2018) and larger spatial distortion near air-tissue interfaces. These complicate co-registrations, and any misregistration can adversely affect multi-contrast segmentation efforts (Peerlings et al., 2019).

Multi-contrast segmentation methods developed for lower field strengths, such as FreeSurfer (Fischl et al., 2002) and Classification using DErivative-based Features (C-DEF) (Selvaganesan et al., 2019), may be degraded in their effectiveness when applied to 7T MRI data (Spini et al., 2020). FreeSurfer is a widely used probabilistic atlas-based segmentation tool that performs well on normal appearing brain scans. C-DEF has been shown to outperform FreeSurfer's segmentation in cases with high MS lesion load or brain atrophy but relies on AFNI tools for skull stripping (Cox, 1996). Instead of relying on an atlas, C-DEF uses a logic regression model trained on image features extracted from MRI intensity derivatives to classify tissue types (Selvaganesan et al., 2019). Fluid-attenuated inversion recovery (FLAIR) images are typically valuable for detecting lesions at 3T and lower field strengths. However, they are less useful at 7T due to generally less lesion conspicuity and stronger bias fields from multiple RF pulses (Zwanenburg et al., 2010; Spini et al., 2020).

Deep learning (D/L) methods, especially convolutional neural networks (CNNs) derived from the U-Net framework, have become state-of-the-art for brain MRI segmentation in recent years (Svanera et al., 2021). These methods rely on layers of automatically optimized filters and nonlinear activations that enable learning sophisticated image features from an annotated training dataset, which are then applied to predict on unseen data. A few prior studies have attempted to apply CNNs to segment high-field MRI data. Custom CNNs have been used for cortical lesion segmentation (La Rosa et al., 2021) and multi-class whole-brain segmentation (Svanera et al., 2021) on 7T data with varying success. Recently, the nnU-Net method introduced by Isensee et al. (2021) has become the *de facto* baseline segmentation method for a wide range of medical segmentation tasks. The nnU-Net is a self-configuring, deep learning framework designed to tackle image segmentation tasks from diverse biomedical datasets. The algorithm follows a standard U-Net architecture (Ronneberger et al., 2015) consisting of an encoder and a decoder which are linked by skip connections. The nnU-Net extracts dataset properties, such as image size, voxel spatial information, and category proportion, to automatically tune hyperparameters, guiding the construction and data manipulation of the neural network. After 5-fold cross-validation, nnU-Net will select the model with best configuration of overall performance from three different U-Net configurations: a 2D U-Net, a 3D U-Net running at full image resolution, and a 3D U-Net cascade. We therefore hypothesized that the capabilities of nnU-Net, perhaps boosted by domain-specific adaptation, may reduce the dependence on auxiliary image contrasts or a *priori* information by relying instead on contextual information extracted from the training dataset.

This manuscript presents a deep learning method for atlas-free, automatic, skullstripping and whole-brain segmentation of 7T MRI, integrating the interdependent tasks of skull stripping and lesion segmentation. The novel method, Pseudo-Label Assisted nnU-Net (PLAn), entails pre-training an nnU-Net model with readily obtained pseudo-label data derived from scans at a lower field strength (3T), then fine-tuned with limited 7T expert-drawn labels (transfer learning) to optimize lesion segmentation performance. The performance of this method was compared against commonly available methods on 3T and 7T data and manually drawn lesion masks in patients clinically diagnosed with MS.

## Methods and materials

The Natural history of MS study protocol was approved by the institutional review board of our institute (NCT00001248), and all participants provided written informed consent.

### Cohorts

In this retrospective study, 33 MS patients were included in two cohorts to train and evaluate nnU-NET, PLAn, and C-DEF-7T. Cohort 1 consists of 25 patients (15 with relapsing-remitting MS, 8 with secondary progressive MS, 1 with primary progressive MS, and 1 with MS-mimicking brain lesions). Inclusion criteria for Cohort 1 was available 3T and 7T scans within 9 months of each other; no clinical disease progression or new lesions between their 3T and 7T scans (median time between 3T and 7T scan: 36 days, range: 1–196 days). 3T and 7T scans from this cohort will be referred to as Cohort 1-3T and Cohort 1-7T. Cohort 2 included 8 MS patients (5 with relapsing-remitting MS, 1 with secondary progressive MS, and 2 with clinically isolated syndrome) scanned only at 7T (mean age: 60.5 years, female = 4), and was mainly used to validate the segmentation technique.

### MRI acquisition

3T images, acquired on a Skyra system with a 32-channel head coil, included 3D T1-weighted (T1w) images (MP2RAGE, TR/TE/TI1/TI2 = 5,000/2.26/700/2,500 ms, flip angle = 4.5°, 1-mm isotropic resolution), 3D FLAIR (TR/TE/TI = 5,000/393/1,800 ms, 1-mm isotropic resolution), and 2D PD/T2 (FSE, TR/TE1/TE2 = 3,630/9.6/96 ms, resolution = 0.7 × 0.7 × 3 mm). 7T images were acquired on a Magnetom system equipped with a 1-channel transmit/32-channel receive coil (Nova Medical and included T1w images) and T_1_ maps (MP2RAGE, TR/TE/TI1/TI2 = 4,000/4.6/350/1,350 ms, flip angle = 4, 0.5°, 0.7-mm isotropic resolution).

### Reference masks for training and validation

Cohort 1 was randomly split into a training/validation group (*n* = 5) and a testing group (*n* = 20). Segmentation labels for training/validation were created for cohort 1-3T using C-DEF as previously described (Selvaganesan et al., 2019). For cohort 1-7T, segmentation labels for training/validation were created using FreeSurfer on MP2RAGE T1w image as a first pass for gray matter (GM), white matter (WM), and cerebrospinal fluid (CSF). These masks were then heavily edited for errors, and a manually drawn lesion mask and skull stripping mask were added to it (C.D, H.D). The final brain segmentation masks were validated by trained and experienced neurologists (E.S.B or M.I.G). For cohort 2, manual lesion segmentations were drawn on the MP2RAGE T1w image using ITK-Snap (C.D) and validated by a neurologist (E.S.B, M.I.G).

### Segmentation algorithms

Reference volumes to evaluate whole-brain and lesion segmentations quantitatively were obtained using C-DEF on 3T scans (referred to as C-DEF 3T) as described elsewhere (Selvaganesan et al., 2019). This was done to allow for detailed comparison and a reliable reference point without large-scale manual annotation. Briefly, the method involves using multi-contrast MRI images (MP2RAGE: uniformized-denoised T1w, inversion 1, inversion 2 and FLAIR), registered and bias-field corrected using a sliding percentile filter. This algorithm generates image features using Gaussian blur and Gaussian gradient filters of various kernel sizes. These features are then used to train a logistic regression classifier with L2 regularization. This was followed by 5-fold cross-validation on the manually annotated training/validation group and ensembled inference using majority voting.

C-DEF segmentations were obtained from 7T MP2RAGE images (T_1_ map, inversion 1, and inversion 2) as well for comparison (C-DEF 7T). The only changes made to the original pipeline were that the percentile filter bias correction (Vovk et al., 2011; Selvaganesan et al., 2019) was omitted in favor of the N4 bias correction (Tustison et al., 2010), and per-image z-score normalization was applied during preprocessing.

FreeSurfer segmentations on 7T (FreeSurfer 7T) were obtained by down-sampling 7T T1w MP2RAGE scans to 1.0 mm^3^, processing by *recon-all*, then up-sampling back to 0.7 mm^3^ space. FreeSurfer automatic subcortical segmentation outputs were converted into NIfTI format, then mapped from various anatomical labels to one of four tissue classes: CSF, GM, WM, and lesions.

An nnU-Net segmentation model (nnU-Net 7T) was trained using cohort 1-7T's training/validation group (*n* = 5) utilizing the publicly available nnU-Net package (Isensee et al., 2021) with the T_1_ map, inversion 1, and inversion 2 images from 7T MP2RAGE scan as inputs. nnU-Net 7T segmentations were obtained after running the full cross-validation, model selection, and ensembled inference routine as described in the nnU-Net documentation (https://github.com/MIC-DKFZ/nnUNet). The only significant modification made to nnU-Net 7T was to disable largest-connected-component post-processing, which was not helpful for this task.

Finally, a 3T to 7T transfer learning model was trained for 7T segmentation (PLAn 7T). The C-DEF 3T model segmentation labels (referred as pseudo-labels for this purpose) and 3T MP2RAGE scans were used to pre-train an nnU-Net model using the 3D full-resolution U-Net configuration and default settings. Transfer learning was achieved by loading the pre-trained model weights and preprocessing settings (except for the final softmax layer), then fine-tuning the network with the manually edited 7T labels and 7T MP2RAGE scans from cohort 1-3T training group (*n* = 5). Fine-tuning was conducted for 125 epochs (default = 1,000) as preliminary analysis found it to be the shortest training regime that remained stable. The initial learning rate was 1 × 10 – 4 (default = 1 × 10 – 2), which was determined by cross-validation to be the best compromise between preserving the pre-trained weights and learning new information from the 7T data. No other settings were altered, and no model layers were frozen during fine-tuning. The fine-tuned model was then applied to the 7T MP2RAGE scans of the remaining unseen participants to obtain cross-validation results.

### Evaluation of segmentation methods

For qualitative analysis, segmentation outputs were randomly scrambled, and the method to generate each segmentation method was hidden. Two experienced neurologists (M.I.G, H.T) rated the quality of segmentation for each tissue class and skull stripping for each participant scan. A subjective rating scale from 1 to 5 was used, with the best segmentation being ranked as a 5 (Table 1). The mean tissue scores and 95% confidence interval from all participants for each method was then calculated and compared.

**Table 1:** Criteria for subjective rating scale used to rank tissue segmentation.

**Rating**	**White matter lesion**	**WM/GM/CSF/skull-stripping**
1	0–25% segmented, 100–75% not segmented or segmented as other structure	Most tissue misclassified
2	25–50% segmented, 75–50% not segmented or segmented as other structure	Tissue class defectively segmented, substantially misclassified
3	50–75% segmented, 50–25% not segmented or segmented as other structure	Tissue is mostly segmented, and scarcely misclassified
4	75–100% segmented, 25–0% not segmented or segmented as other structure	Tissue is completely segmented, and scarcely misclassified
5	All lesions segmented, well defined and matching the exact shape of the lesions	Tissue is mostly segmented, without misclassification

An extensive quantitative analysis was performed on volumetric agreement between C-DEF-3T segmentation outputs and the 7T segmentation algorithms on the 20 participants from cohort 1's testing group. Tissue volumes were calculated using the *fslstats* utility from FSL (Jenkinson et al., 2012). Skull stripping between all methods was also evaluated using Bland-Altman analysis to determine mean bias and 95% limits of agreement between methods, as it has large implications in tissue volumes. Importantly, C-DEF 3T and C-DEF 7T use AFNI's 3dSkullStrip tool (shrink_fact_bot_lim = 0.7, AFNI toolkit) (Cox, 1996) on the 3T (AFNI 3T) and 7T (AFNI 7T) MP2RAGE second inversion images. Relationships between tissue segmentation volumes between methods were calculated using Pearson's correlation coefficient and Dice similarity coefficient (DSC) was calculated for each tissue class as previously described (Zou et al., 2004) with C-DEF 3T reference images. Finally, PLAn 7T and nnU-Net 7T lesion segmentation was further evaluated by comparing volumes to manually drawn lesion segmentations in cohort 2.

Statistical analysis was performed using PRISM version 9.3.1. After checking the normality assumption using the Shapiro-Wilk test, we employed repeated-measures ANOVA using Dunnett's method to compare mean metric differences of segmentation methods to either the 3T reference or PLAn (as appropriate), corrected for multiple comparisons. A corrected *p*-value of 0.05 or lower was considered statistically significant. Unless otherwise noted, all statistics are given in terms of their mean value and 95% confidence intervals (CI), and all data reported are in the form of mean ± standard deviation.

## Results

### Skull stripping

**Figure 1:**
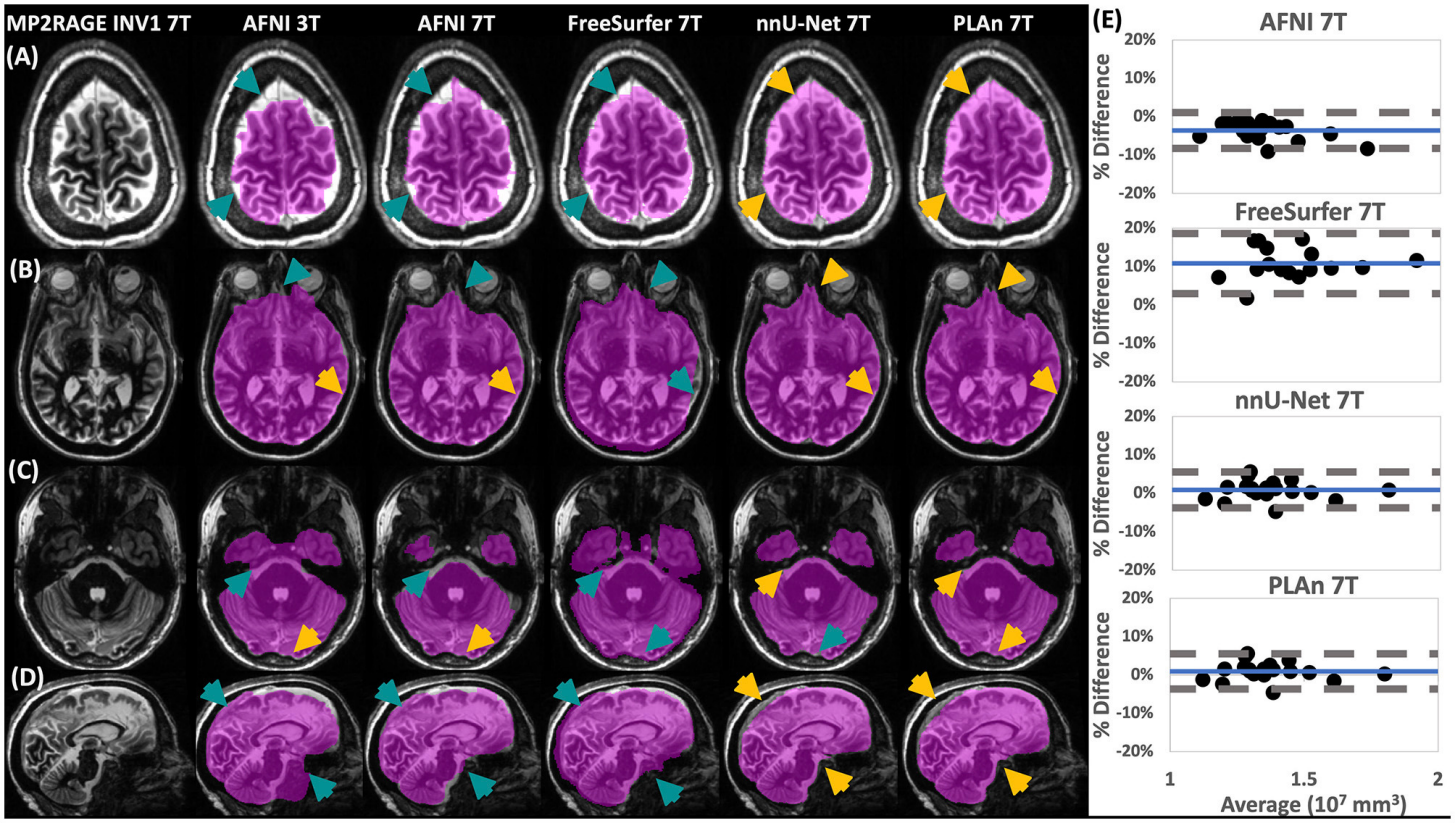
Qualitative and quantitative assessment shows nnU-Net and PLAn methods give superior skull stripping results at 7T. At left, representative **(A–C)** axial and **(D)** sagittal image slices selected from various participants from the inference set show the output of skull stripping methods (magenta overlay indicates regions identified as brain, method and field strength indicated at the top) in various brain regions overlaid on 7T MP2RAGE INV1 image. Gold arrows indicate good skull stripping features in areas where other methods made errors (teal arrows). At right, **(E)** Bland-Altman plots of total intracranial volume (TIV) calculated from 7T methods in **(A–D)** compared to AFNI 3T, which was used as a reference (% Difference = 100 x [Method – C-DEF 3T]/Average). Mean bias (solid blue) and 95% limits of agreement of the dataset (dashed gray) are indicated.

First, skull stripping performance was evaluated using qualitative ratings and Bland-Altman plots, which compared total intracranial volumes (TIV) to the reference AFNI 3dSkullStrip performed on 3T MP2RAGE (AFNI 3T). The nnU-Net 7T and PLAn 7T methods produced excellent skull stripping results throughout the brain (Figures 1A–D). Bland-Altman analysis (Figure 1E) showed that both methods had very low mean TIV biases, with 0.88% (CI: −3.8, 5.6) for nnU-Net 7T and 0.89% (CI: −3.7, 5.5) for PLAn 7T. The qualitative evaluation revealed that deep learning methods offered slight improvements over AFNI at 7T, particularly in dorsal (Figure 1A), retro-orbital (Figure 1B), and cerebellum (Figure 1C), and near the superior sagittal sinus and brainstem (Figure 1D) regions. Indeed, Bland-Altman analysis comparing AFNI 7T to AFNI 3T (Figure 1E) produced a mean TIV bias of −3.5% (CI: −8.2, 1.1). Expert ratings (Table 2) confirmed these observations, with mean skull stripping scores of 4.42 (CI: 4.33, 4.51) for nnU-Net 7T and 4.4 (CI: 4.3, 4.5) for PLAn 7T, respectively, compared to 4.03 (CI: 3.7, 4.2) for AFNI 7T skull stripping.

**Table 2:** Blinded expert ratings (mean with 95% CI) for each 7T method for skull stripping and tissue segmentation.

	**FreeSurfer 7T**	**C-DEF/AFNI 7T**	**nnU-Net 7T**	**PLAn 7T**
Skull stripping	1.82 [2.07, 1.56]	4.03 [4.2, 3.86]	4.42 [4.51, 4.33]	4.4 [4.50, 4.30]
Cerebrospinal fluid	1.58 [1.86, 1.3]	2.76 [2.98, 2.55]	3.89 [4.08, 3.7]	3.9 [4.09, 3.71]
Gray matter	1.95 [2.14, 1.75]	2.39 [2.63, 2.16]	3.34 [3.5, 3.18]	3.39 [3.55, 3.24]
White matter	2.5 [2.79, 2.21]	3.16 [3.39, 2.93]	3.92 [4.01, 3.83]	3.92 [4.01, 3.83]
Lesions	1.95 [2.22, 1.67]	1.63 [1.90, 1.37]	3.74 [3.98, 3.49]	3.92 [4.16, 3.68]

Rating scale is from 1 being poor and 5 being accurate segmentation for each class.

While AFNI 7T and AFNI 3T produced similar skull stripping boundaries in some brain regions, such as dorsal regions (Figure 1A), other regions, such as the cerebellum, showed significant degradation at 7T compared to 3T (Figure 1C). FreeSurfer 7T, on the other hand, was prone to overestimation of intracranial volumes, including extracranial areas abutting the anterior temporal lobe (Figure 1D) and cerebellum (Figure 1C). This resulted in a mean TIV bias of 10% (CI: 3, 19) (Figure 1E). These errors were more detrimental than those displayed by AFNI 7T, as expert ratings of skull stripping quality produced a mean score of just 1.82 (CI: 1.6, 2.1) for FreeSurfer 7T (Table 2).

### Comparison of tissue segmentation methods

**Figure 2:**
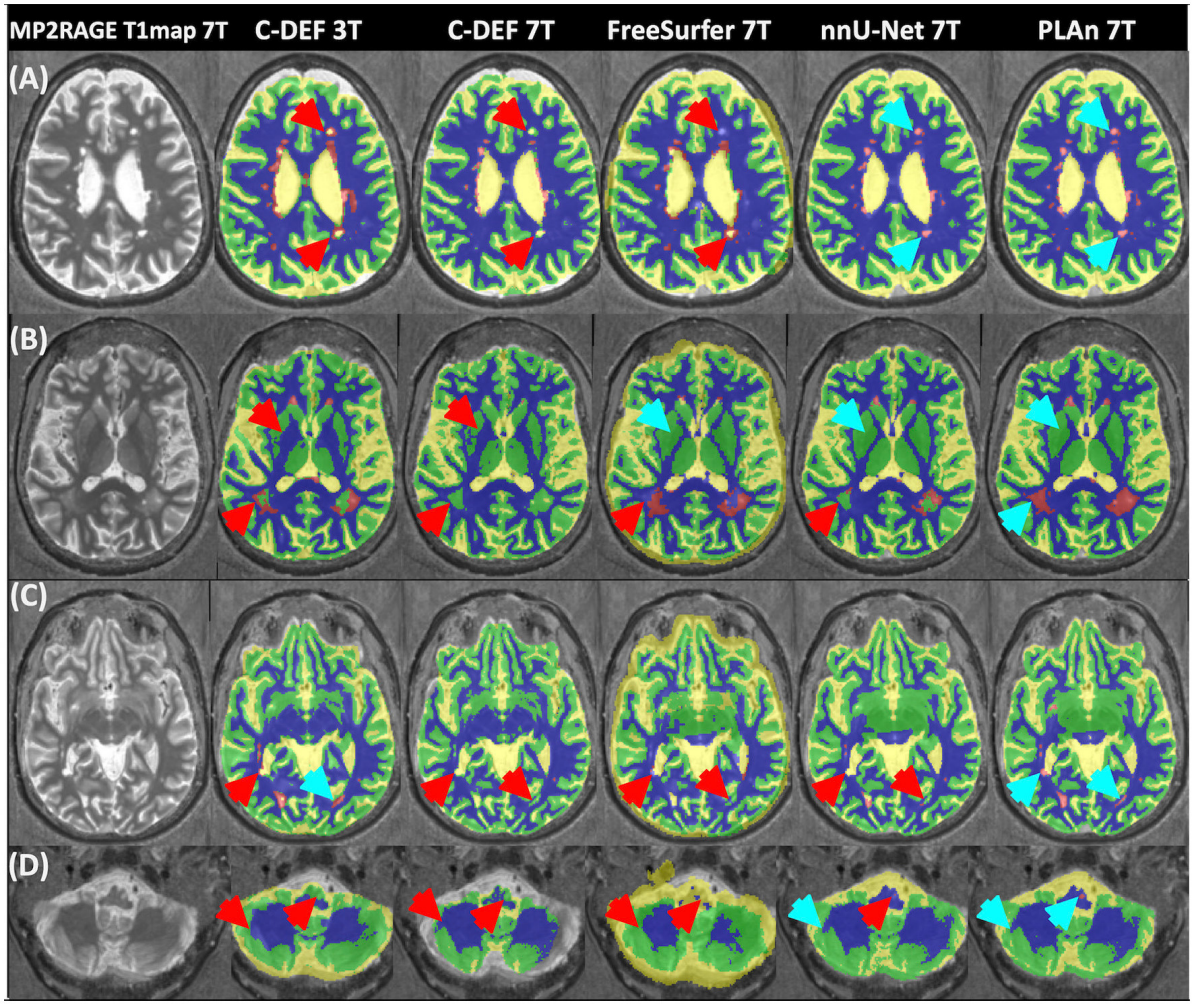
Qualitative assessment shows that PLAn 7T produces the best overall segmentations at 7T. Representative axial image slices selected from various participants in the inference set show different tissue segmentation methods (method and field strength indicated at the top) from various **(A–C)** supratentorial, and **(D)** infratentorial brain regions overlaid on 7T MP2RAGE T_1_map image. Blue arrows indicate good segmentation features, red arrows indicate errors. For the segmentation overlays, blue indicates WM, yellow CSF, green GM, and magenta lesions.

**Figure 3:**
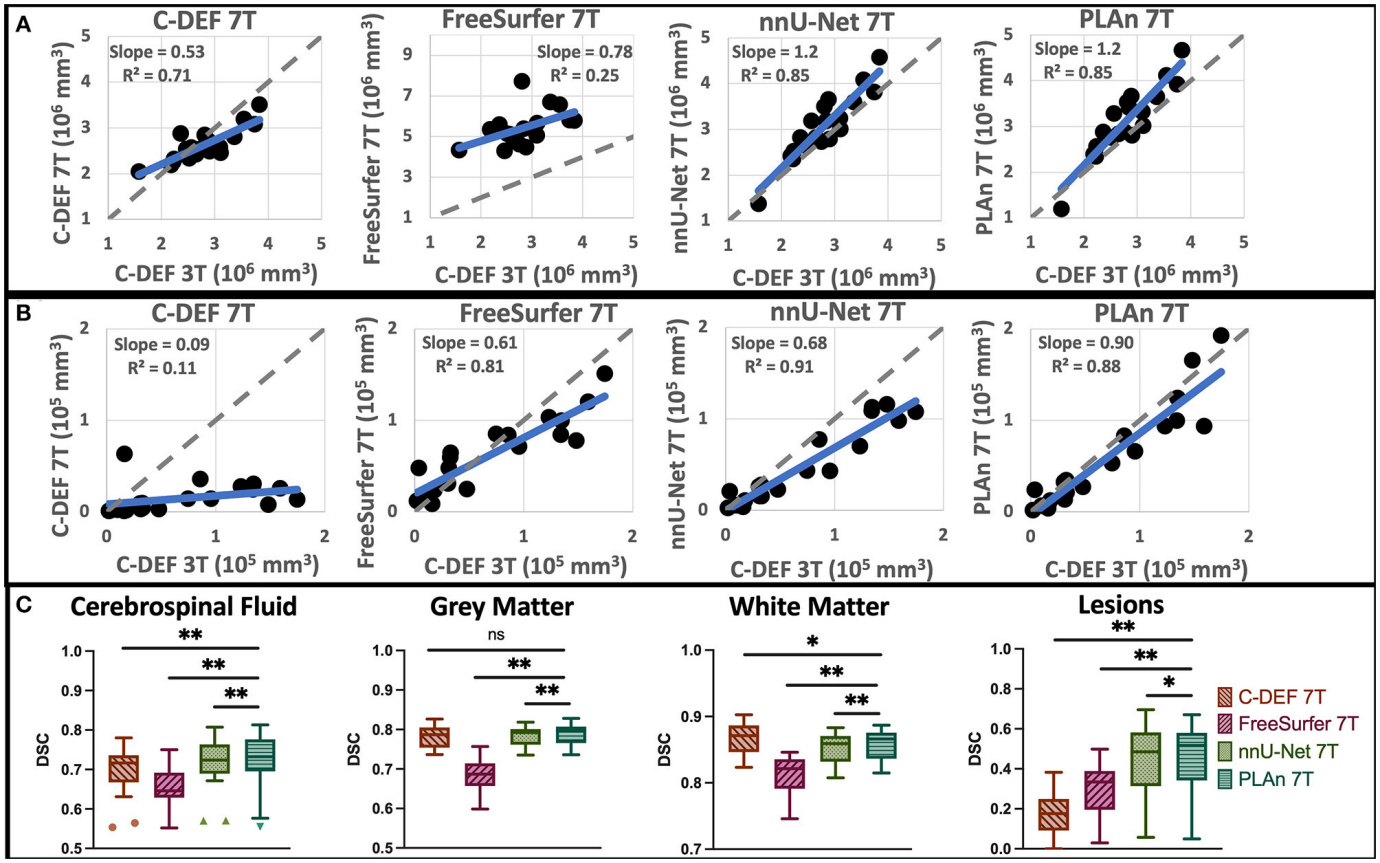
Quantitative assessment shows that PLAn 7T produces the best overall segmentations at 7T. Correlation plots of **(A)** CSF volume and **(B)** lesion volume of various segmentation methods (as indicated on top) vs C-DEF 3T reference with identity (dashed gray) and regression (solid blue) lines. **(C)** Dice similarity coefficient (DSC) plots of each tissue class vs C-DEF 3T reference; asterisks mark significant mean difference compared to PLAn 7T (*p < 0.05, **p < 0.01).

All volumetric comparisons are in reference to the 3T segmentation outputs. The D/L methods (nnU-Net 7T and PLAn 7T) significantly outperformed both C-DEF 7T and FreeSurfer 7T for all tissue segmentation classes from cohort 1 (Figure 2). They successfully captured detailed cortical boundaries (Figures 2A, B) and white matter lesions (Figures 2A–C) as well as cerebellum and brainstem borders (Figure 2D). Cerebrospinal fluid (CSF) volume from D/L methods showed the strongest correlations with C-DEF 3T (Figure 3A, *r* = 0.92, CI: [0.91, 0.97], *p* < 0.001, slope = 1.2). This is consistent with prior observations that C-DEF 3T under-strips the skull in dorsal regions, leading to slightly lower CSF volumes than expected. FreeSurfer 7T produced degraded temporal lobe and cerebellum segmentation, mislabeling sulcal gray matter (GM) as CSF throughout the ventral regions (Figures 2C, D). This resulted in highly inflated and unreliable CSF volumes, with a mean bias of 63% (CI: 30, 96) along with a comparatively low correlation coefficient (*r* = 0.50, CI: [0.02, 0.79], *p* > 0.05, slope = 0.78). C-DEF 7T had much better CSF segmentations due to good cortical boundaries (Figures 2A, B), although it still presented a modest mean CSF volume bias of −4.7% (CI: −29, 20). While this bias did have a significant effect, resulting in a mean CSF DSC of 0.70 (CI: 0.67, 0.73), compared to 0.72 (CI: 0.69, 0.75) for nnU-Net 7T, it was less detrimental than the errors in FreeSurfer 7T (mean DSC: 0.65, CI:[0.63, 0.68]). Expert ratings confirmed that nnU-Net was the best 7T method for CSF segmentation, with a mean rating of 3.89 (CI: 3.7, 4.08), compared to 1.58 (CI: 1.3, 1.86) for FreeSurfer 7T and 2.76 (CI: 2.55, 2.98) for C-DEF 7T (Table 2). The nnU-Net also matched or exceeded FreeSurfer 7T in distinguishing deep GM structures such as the thalamus and globus pallidus, which C-DEF often missed (Figure 2B). As a result, nnU-Net 7T had substantially better expert ratings than C-DEF 7T or FreeSurfer 7T for both GM and WM (Table 2).

Correlation plots (Figures 3A, B) show a strong correlation between nnU-Net 7T and C-DEF 3T in lesion volumes (*r* = 0.95 CI: [0.89, 0.98], *p* < 0.001, slope = 0.68). C-DEF 7T, on the other hand, failed to detect most lesions throughout the brain (Figures 2A–D), which resulted in very low lesion volumes (Figure 3B) and a correlation of just *r* = 0.33 (CI [−0.13, 0.68], *p* > 0.05, slope = 0.09). Meanwhile, FreeSurfer 7T demonstrated significant lesion sensitivity, with a moderately strong lesion volume correlation of *r* = 0.90 (CI: [0.75, 0.96], *p* < 0.001, slope =0.61), both of which were significant increases compared to C-DEF 7T (Figure 3B). However, qualitative inspection revealed these lesion segmentations to be frequently inaccurate (Figures 2A–C). Lesion volumes of participants with low lesion loads were inflated, whereas those with high lesion loads were deflated (Figure 3B). Both lesion DSC (mean: 0.30 [CI: 0.23, 0.36]) and expert ratings of 1.95 (CI: 1.67, 2.22) in FreeSurfer 7T were only slightly better than that of C-DEF 7T, despite far greater lesion volumes.

### PLAn 7T improves lesion segmentation over nnU-Net 7T

Despite its substantial advantages over other 7T methods, nnU-Net 7T still produced noticeable deficiencies in lesion detection (Figures 2B, C). To address these deficiencies, we implemented the PLAn 7T method. Using cohort 1's testing group, mean DSC values calculated against C-DEF 3T indicated small, but statistically significant improvements in CSF (1%), GM (0.6%), and WM (0.5%) segmentation in PLAn 7T compared to nnU-Net 7T. Expert ratings (Table 2) found either no (CSF, WM) or slight (GM, 1.5%) improvements for tissue segmentation using PLAn 7T compared to nnU-Net 7T. Correlation plots of CSF volumes vs C-DEF 3T (Figure 3A) also appeared virtually identical between the two methods. PLAn 7T gave a consistent boost to lesion volumes (mean bias: 15%, CI: [−25, 55]) compared to nnU-Net 7T. This resulted in the slope of the regression line increasing significantly (*p* < 0.05) from 0.68 for nnU-Net 7T to 0.90 for PLAn 7T and corresponded to an improved expert lesion rating of 3.92 (CI: 3.68, 4.16) for PLAn 7T, compared to 3.74 (CI: 3.49, 3.98) for nnU-Net 7T (a 5% increase).

**Figure 4:**
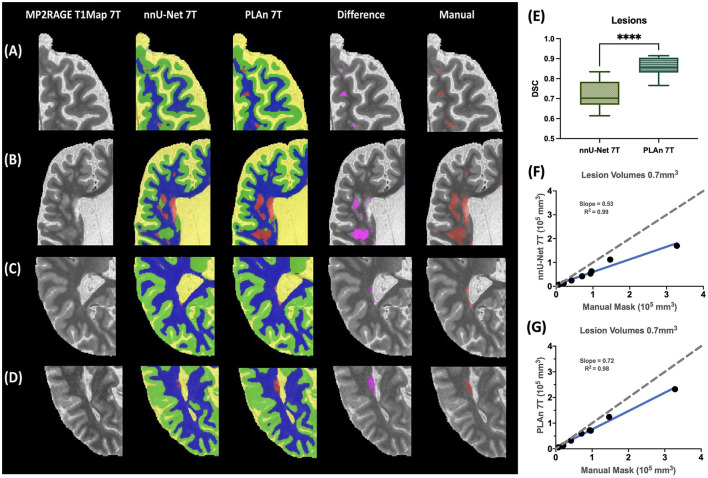
Improved lesion segmentation in PLAn 7T compared to nnU_Net on unseen cohort. Detailed comparison of segmentation results from nnU-Net (column 2) and PLAn (column 3) from various participants highlighting **(A)** punctate lesions in frontal lobe, **(B)** periventricular and deep white matter lesions, and **(C, D)** lesions in occipital horn of lateral ventricle in color, overlaid on 7T MP2RAGE T_1_ map. Voxelwise subtraction of the lesion mask from nnUNet 7T and PLAn 7T methods is overlaid in pink on the T_1_ map (column 4). Manual lesion segmentation is overlaid in red on T1 map (column 5). **(E)** Dice similarity coefficient (DSC) of selected methods calculated from lesion segmentation masks vs manual segmentation mask (^****^p < 0.0001). Correlation of lesion volume from nnU-Net and **(F)** PLAn, and **(G)** manual segmentation mask.

The significant improvement in PLAn 7T's lesion sensitivity compared to nnU-Net 7T was validated in an unseen cohort (Cohort 2, Figure 4). The improved segmentation was consistent across various lesion types, including diffuse WM hyperintensities that nnU-Net 7T was often unable to distinguish from GM or WM (Figures 4A, B), as well as periventricular lesions, which were often misclassified as CSF (Figures 4C, D). Quantitative analysis of 7T lesion segmentation revealed significantly higher DSC (Figure 4E) in PLAn 7T (mean = 0.86, CI: [0.83, 0.90]) than nnU-Net (mean = 0.72, CI: [0.78, 0.67], *p* < 0.0001) when compared to C-DEF 3T. In addition, PLAn showed stronger slope which was closer to unity (dotted line in Figures 4F, G) (slope = 0.72) than nnU-Net (slope = 0.53) with C-DEF 3T indicating it had less systemic underestimation at higher lesion volumes.

## Discussion

This study presents PLAn, a novel automatic whole-brain segmentation, including lesion segmentation, using a single, commonly gathered MRI sequence at 7T. The PLAn tool integrates skull stripping and whole-brain segmentation including lesion segmentation in a single step. This enables simpler and faster training, domain adaptation, and inference than previous methods in which these steps are handled by separate models. We show that nnU-Net, a publicly available medical image segmentation method, can produce accurate segmentations even with a few well-annotated training examples, outperforming existing skull stripping and tissue segmentation methods. Adding a pre-training step to nnU-Net-7T (in PLAn-7T) significantly improved lesion segmentation performance by leveraging the readily available 3T pseudo-labels to mitigate limited 7T label availability. Importantly, PLAn-7T only requires a 3T cohort for training, but not in application of the segmentation.

For all segmentation tasks at 7T, D/L recon methods clearly outperformed C-DEF and FreeSurfer. nnU-Net's excellent skull stripping is consistent with prior studies which established the efficacy of U-Net-derived CNNs for brain extraction in general (Hwang et al., 2019; Wang et al., 2021) and in MS patients (de Oliveira et al., 2022). In contrast, FreeSurfer and AFNI were prone to under or over-skull stripping. This was particularly detrimental to FreeSurfer's downstream segmentation tasks, in which the poor skull stripping dramatically reduced CSF segmentation quality. Notably, nnU-Net integrated skull stripping and segmentation into a single step.

Overall, nnU-Net 7T effectively combined the advantages of C-DEF 7T (good CSF segmentation and cortical boundaries) and FreeSurfer (detailed GM segmentation). Nevertheless, FreeSurfer's cortical segmentations in the temporal lobe and cerebellum suffered from degradation. Given this degradation's localized and systematic nature, it is likely due to inadequate mitigation of the strong 7T bias fields, as previously found in FreeSurfer analyses at ultra-high fields (Haast et al., 2018). Indeed, sliding percentile filter based bias correction that was employed at 3T was found to be insufficient for correcting the bias fields at 7T, and it was also found to exasperate artifacts due to susceptibility effects. On the other hand, N4 bias field correction has worked particularly effective in addressing these challenges at 7T. PLAn 7T produced similar high-quality segmentation results to the default nnU-Net 7T. Despite AFNI's skull stripping errors at 3T, PLAn 7T produced excellent results, suggesting that the fine-tuning effectively corrected errors in the 3T pseudo-labels. It also retained the other aspects of the 7T baseline model that were already optimal, including deep GM structures and cortical details.

PLAn 7T's pre-training step boosted nnU-Net's lesion sensitivity. nnU-Net, while more effective at lesion detection than the other baseline methods, still had substantial room for improvement. With reference to manually drawn lesion masks, PLAn's DSC improved by 16% compared to nnU-Net. PLAn dilated existing lesion boundaries to cover the edges of diffuse WM hyperintensities, frequently added missing chunks of large diffuse lesions, or detected lesions missed by all other methods (Figures 2, 4).

Prior studies indicate that transfer learning is often advantageous when the training dataset in the target domain is small (Valverde et al., 2021). In this study, nnU-Net produced superior lesion segmentation compared to commonly used methods at 7T with only five training labels. This is a far smaller training dataset than typically used for CNN training, even for MRI segmentation tasks, for which manually validated annotations are highly time-consuming and expensive to generate. In PLAn, we leveraged readily available data from a lower field strength to pre-train the segmentation model. Pre-training the network on a larger, similar dataset allowed for more examples of lesion morphology and improved lesion segmentation. C-DEF 3T inference segmentations were chosen as pseudo-labels for the pre-training step since they were readily available and reasonably accurate, as demonstrated previously (Selvaganesan et al., 2019; Dieckhaus et al., 2022), although not entirely free from errors.

FLAIR contrast is invaluable for distinguishing lesions from GM and CSF. Prior studies have found that 3T FLAIR images can indicate more accurate lesion boundaries than those visible on 7T or 3T MP2RAGE scans. When re-trained on 7T MP2RAGE scans, PLAn was able to recognize subtle lesion indicators that were associated with 3T FLAIR hyperintensities, even in the absence of a 7T FLAIR, which likely contributed to its ability to detect diffuse and periventricular lesions better. The lack of FLAIR contrast at 7T may explain why C-DEF 7T failed to detect lesions, unlike its 3T counterpart. At 3T, C-DEF has previously been shown to perform better than a U-Net segmentation algorithm when trained on limited labels (Dieckhaus et al., 2022). In this study, nnU-Net 7T clearly outperformed C-DEF 7T, suggesting that MP2RAGE images alone do not provide sufficient contrast for C-DEF to reliably separate lesions from other tissues, essentially a lack of orthogonal data. Additional contrast might improve the performance of C-DEF 7T, as long as susceptibility/distortion artifacts can be minimized between these sequences. We did evaluate the efficacy of T2^*^-weighted 3D echo-planar imaging scans as a potential independent contrast. However, the images suffered from residual distortion errors near the sinuses and were not further considered. Along with co-registration difficulties at 7T, high-field FLAIR images suffer from increased bias fields and artifacts, which make them difficult to use for intensity-based segmentation tasks. Therefore, 7T FLAIR images were not collected or evaluated in this study.

One limitation of this study is that the methods evaluated were primarily assessed in an off-the-shelf fashion, with no hyperparameter tuning or modification unless explicitly stated. While dedicated fine-tuning of any of these methods may yield improved results for a specific cohort, such extensive optimization is beyond the scope of this study. In addition, we compared 7T segmentation volumes to segmentations produced by C-DEF at 3T to allow for a detailed comparison to a reliable reference point (C-DEF 3T) without large-scale manual annotation, which can be incredibly expensive and tedious. These labels cannot be treated as a perfect 7T gold standard due to the inherent differences between segmentations at 3T and 7T. Additionally, a FLAIR contrast was included at 3T but not 7T. Previous research has shown that lesion segmentation volumes derived from FLAIR contrasts are higher than from T_1_ maps, although the resulting volumes are highly correlated (Spini et al., 2020). This is in line with our results which showed that C-DEF 3T reference lesions had higher lesion volumes than 7T methods that did not use FLAIR. To prevent bias in comparing 7T methods against each other, blinded neurologist ratings were included without reference to 3T.

PLAn was pre-trained on pseudo-labels derived from cohort 1-3T and then initially evaluated on the same participants scanned at 7T (cohort 1-7T). Then, we compared the lesion segmentation performance of PLAn and nnU-Net on an unseen cohort (cohort 2) with reference to manually drawn labels. Future work will apply PLAn on multi-site data, including other disease cohorts, and refining the pseudo-label and gold standard generation method.

One area for further investigation is whether a larger training data set (source or target domain) would significantly improve the segmentation of lesions or other classes. A recent study of transfer learning for subcortical segmentation found that while just a few (1 to 3) images were sufficient for fine-tuning in most cases, smaller structures such as the amygdala and accumbens were most improved by additional data for fine-tuning (Kushibar et al., 2019). Several recent studies have also used automated or semi-automated imperfect training labels (i.e., pseudo-labels) for model pre-training, followed by few-shot fine-tuning (Bermudez et al., 2020; Svanera et al., 2021). These examples typically use larger source domain datasets than the one utilized in this study (*n* = 20), which may indicate that utilizing more pseudo-labeled data for pre-training could also boost performance.

In this study, we developed and applied a state-of-the-art deep learning method to MP2RAGE 7T MR images to obtain fast and reliable whole-brain segmentations. By employing only multi-contrast techniques such as MP2RAGE, it is readily scalable and adaptable to use in various conditions and performs robustly against any registration errors. The performance of 7T lesion segmentation was boosted, and training label scarcity for lesion segmentation was overcome by incorporating a pre-training step using results from a robust 3T segmentation as pseudo-labels before fine-tuning a transfer-learning method. We present these findings as a blueprint for acquiring fast, accurate, and valuable volumetric markers from 7T MRI data for use in clinical and research settings.

## Computation resources

All C-DEF and FreeSurfer processing was implemented on a computing cluster with 64x Intel^®^ Xeon^®^ CPUs with 256 GB RAM running CentOS 7.7. All nnU-Net and PLAn models were implemented on a computing cluster with NVIDIAÒ v100-SXM2 GPUs, each with 32 GM VRAM and 24 Intel^®^ Xeon^®^ CPUs, each with 64 GB RAM.

## Data availability statement

The raw data supporting the conclusions of this article will be made available by the authors, without undue reservation. PLAn software is available at https://github.com/hdieckhaus/PLAn-7T.

## Ethics statement

The studies involving humans were approved by Institutional Review Board of the National Institutes of Health. The studies were conducted in accordance with the local legislation and institutional requirements. The participants provided their written informed consent to participate in this study.

## Author contributions

All authors listed have made a substantial, direct, and intellectual contribution to the work and approved it for publication.
